# Assessment of HIV Infection in HIV Patients Admitted to Pakistan Institute of Medical Sciences, Islamabad, Pakistan

**DOI:** 10.1155/arat/5549074

**Published:** 2025-04-07

**Authors:** Akmal Zubair, Muhammad Ali, Rizwan Munir, Md. Belal Hossain

**Affiliations:** ^1^Department of Biotechnology, Quaid-i-Azam University, Islamabad, Pakistan; ^2^Department of Statistics, The University of Faisalabad, Faisalabad, Punjab, Pakistan; ^3^Department of Education, Hubei University of Education, Wuhan Hi-Tech Zone, Wuhan 430205, Hubei, China; ^4^Department of Plant Pathology, Faculty of Agriculture, Sher-e-Bangla Agricultural University, Sher-e-Bangla Nagar, Dhaka, 1207, Bangladesh

**Keywords:** blood transfusion, drug users, HIV, Pakistan, safe sex, syringes

## Abstract

**Aims:** This study aims to assess the correlation between risky behaviors (unprotected sexual intercourse with multiple partners, men who have sex with men (MSM), and injectable drug users) and HIV infection among patients. The study focus on evaluating risk behaviors associated with HIV transmission among HIV-positive individuals.

**Background:** HIV is an RNA virus that primarily attacks the immune system. Currently, there are 39 million people infected with HIV.

**Methodology:** This study is a cross-sectional analysis involving 67 HIV patients admitted to the Pakistan Institute of Medical Sciences (PIMS) during the years 2023–2024. All patients were interviewed regarding their HIV infection, and various questions concerning risk factors were posed to them.

**Results:** Our research demonstrates a significant prevalence of HIV among unmarried individuals, with a statistical significance of *p* < 0.01. Furthermore, engaging in the high-risk behavior of sharing syringes and needles (standard beta = 0.73) and associating with drug users (standard beta = 0.061) might be considered forms of unsafe practices. There is a strong positive correlation (*r* = 0.867^∗∗^) between drug users and the practice of sharing syringes and needles, which is highly statistically significant (*p* < 0.01). The results indicate a substantial association between drug users, the sharing of needles and syringes, and HIV infection.

**Conclusion:** Drug users often share needles and syringes with other users, which significantly contributes to the outbreak of HIV in society. Unmarried men exhibit a higher prevalence of HIV compared to married men. Increasing public awareness and implementing robust government policies could help reduce the rate of HIV infections.

## 1. Introduction

Pakistan was once considered very susceptible to an epidemic; however, it now has a low seroprevalence of HIV. National AIDS control programs play an important role in managing HIV infection. There are 72,515 HIV cases reported by September 2024 in 72 HIV centers in Pakistan (https://www.cmu.gov.pk). A significant number of individuals engage in unnecessary self-injections without using sterile needles and syringes. In addition, medical equipment often lacks regular sterilization, and blood banks do not possess the necessary technology to screen potential donors effectively. Approximately 33% of the global population of 12.7 million individuals who use injectable drugs are now infected with HIV. Data collected from several countries indicate a significant increase in infection rates among those living with HIV [1]. The primary demographic groups in Pakistan that exhibit a notable incidence of HIV include sex workers and transgender individuals, people who inject drugs (PWIDs), and men who have sex with men (MSM). The overall HIV prevalence in the population is 0.1%, but several key populations exhibit significantly higher rates. Consistent with previous modeling studies, the Pakistani government conducted five rounds of integrated biological and behavioral surveillance (IBBS) from 2005 to 2017 [2]. These rounds indicate a rising prevalence of HIV among all significant populations, with overlapping risk factors likely contributing to this trend. Given that injectable drug epidemics often originate among PWID due to the sharing of injecting equipment, this demographic is a critical focus for preventive initiatives [3]. The HIV prevalence among PWID in Pakistan has increased to 38%, according to surveillance data from 2016/17, compared with an estimated 20% in the early 2000s [4]. The reuse of nonsterile dental and medical devices significantly increases the risk of contracting HIV, even with minimal contact with blood. Viable HIV can survive for more than 2 hours in various environments, particularly those involving compromised immune systems, such as on sharp surfaces, abandoned needles, and syringes [5, 6]. According to the International Burden of Disease Study (IBDS), the prevalence of HIV among individuals with HIV/AIDS increased from 10.8% to 27.2% between 2005 and 2011. Cities in Pakistan with HIV prevalence rates exceeding 40% in this category include Faisalabad (52.5%), D.G. Khan (49.6%), Gujrat (46.2%), Karachi (42.2%), and Sargodha (30.6%) [7]. The HIV prevalence in capital cities such as Lahore is 32.2%, Peshawar 22.9%, and Quetta 8.3%. The most recent IBBS conducted in 2014 revealed a weighted prevalence of 36.6% across all 10 cities in Punjab. Rizwan et al. projected that the number of HIV-positive injectable drug users (IDUs) in Pakistan could potentially reach 68,000 by 2020, with estimates ranging from 46% to 56% in Karachi and 44% to 50% in Lahore. The prevalence among the PWID population in Faisalabad may reach between 65% and 75% [8]. In Pakistan, obtaining a comprehensive history of HIV patients presents a significant challenge. There is a low likelihood that HIV patients is willing to provide accurate historical information. By analyzing their medical history and risky behaviors, we were able to determine how these patients infected with HIV virus. To date, no study in Pakistan has successfully gathered and analyzed accurate historical data from HIV patients. This is the first study aimed at understanding the behaviors of individuals and the potential factors contributing to HIV infection among these patients.

This aims to evaluate HIV patient's behaviors and routine activity that led to HIV infections. The basic hypothesis of the research is whether risky behavior, such as unprotected sexual intercourse with multiple partners, MSM, IDUs, or working as a sex worker, could possibly spread HIV in Pakistan. Based on their past activities and data shared by HIV patients, we were able to analyze the potential modes of HIV transmission among those infected. The questionnaire is designed to gather comprehensive information about HIV-infected patients and the circumstances surrounding their infection.

## 2. Material and Methodology

### 2.1. Study Site

The study used a cross-sectional self-reported survey design, using questionnaires to assess individuals' level of knowledge of HIV and its modes of transmission. The study was conducted at the Pakistan Institute of Medical Sciences (PIMS) Hospital between 2023 and 2024. PIMS is the largest government-operated hospital in Pakistan and has a specialized unit for HIV treatment. The Pakistan National AIDS Control Program collaborates with PIMS Hospital and the National Institutes of Health (NIH) Pakistan. The demographic data of individuals infected with HIV is represented in Table 1.

All the HIV-positive male patients were included in Pakistan and HIV-negative patients were excluded from the study. Most questions related to males such as beard shaving in saloons, male homosexuality, and IDUs. All of the females and their family were agree to participate in the study but the questionnaires were not related to females so they were excluded from the study.

### 2.2. Sample

**Figure 1:**
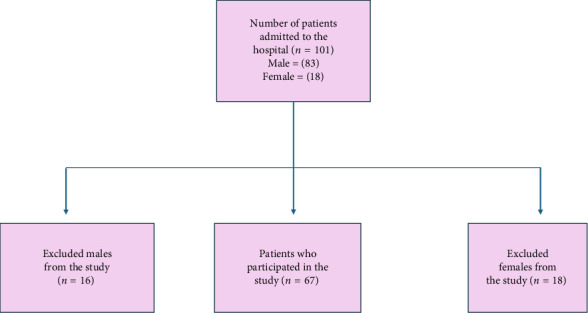
The flowchart of patients admitted in the hospital and the number of patients excluded from the study.

During the 2023-2024 period, the PIMS hospital admitted 102 individuals diagnosed with HIV. Of these, 67 patients consented to participate in the study, while 35 patients declined to participate in any capacity. The questionnaire performance was not related to females; so they were not included in the study. All HIV-infected patients admitted based on their viral load and CD4 levels were included in the study cohort.  Figure 1 illustrates the HIV-positive patients who participated in the study.

### 2.3. Instrument and Questionnaire Performa

An extensive number of validated questionnaires were used in previous research on this topic (1–5). These surveys served as the foundation for the items that were later incorporated into the self-administered questionnaire used for this investigation. To ensure the questionnaire contained valid information, it was reviewed by four medical professionals with expertise in infectious diseases, specifically HIV/AIDS. After evaluating the content of the questionnaire and implementing any necessary modifications, we conducted an initial test to assess its reliability. The validated questionnaire comprised four sections: demographics, history of drug use, history of transfusions, and history of sexual activity. The questionnaire was developed in both English and the national language (Urdu) to enhance comprehension of each question. In addition, each question was explained in the local language to facilitate understanding.

A set of 17 questions was employed to assess the respondents' familiarity with HIV/AIDS. The investigation covered a broad spectrum of topics, including safe sexual practices, drug use, needle sharing, and blood transfusions, among others. For the knowledge component, each question offered three response options: “yes,” “no,” and “do not remember.”

### 2.4. Statistical Analysis

To conduct statistical analysis, all information gathered from the questionnaires was initially entered into Microsoft Excel and subsequently uploaded to SPSS for Windows Version 19 (SPSS Inc., Chicago, Illinois, United States).

### 2.5. Ethics

To proceed with the investigation, permission was obtained from the Ethics Committee of Quaid-i-Azam University Islamabad.

## 3. Results

Table 2 presents a comparison of various factors, including blood transfusion, unsafe sexual practices, sharing syringes and needles, and drug use. The *p* value of < 0.05 for the variables related to blood transfusion, sharing syringes and needles, and drug use indicates no significant association between married and unmarried HIV patients. However, a significant association is observed in the context of unsafe sexual practices, as indicated by a *p* value of < 0.005. The same four variables were also analyzed by region. No significant associations (*p* > 0.05) were found in Azad Jammu and Kashmir (AJK), Gilgit-Baltistan, Khyber Pakhtunkhwa (KP), Punjab, and Baluchistan. In addition, at the educational level, there is no significant association between education (including primary, high school, no schooling, and F.Sc.) and the four variables: blood transfusion, unsafe sexual practices, sharing syringes and needles, and drug use.

The Pearson correlation coefficient indicates a linear relationship between blood contamination, unsafe sex, sharing syringes and needles, and drug users, as shown in Table 3. A weak negative correlation is present between blood contamination (−0.086), while weak positive correlations are found between sharing syringes and needles (0.138) and drug users (0.130). The *p* value indicates that there is no significant correlation. Unsafe sex exhibits a weak negative correlation with blood transfusion (−0.086). In addition, there is a weak positive correlation between unsafe sex and sharing syringes and needles (0.073) as well as drug users (0.061). A strong positive correlation exists between drug users and sharing syringes and needles (0.867^∗∗^), and this correlation is highly significant (*p* < 0.01). Furthermore, a weak positive correlation is observed between drug users (0.138) and blood transfusion, as well as sharing syringes and needles (0.073). According to Table 2, there is a statistically significant difference (*P* < 0.001) in the frequency of risky sex among married and unmarried adults. Drug usage, sharing syringes, and blood transfusions were not substantially different by location, education level, or marital status. The connection between sharing syringes and drug usage was very significant (*r* = 0.867, *p* < 0.001) in Table 3. The other habits, however, did not show any significant correlations.

### 3.1. Regression Analyses Factors Associated With Blood Transfusion, Unsafe Sex, Sharing Syringes and Needles, and Drug Users Regarding HIV

Preliminary linear regression analysis uses the blood transfusion score as the dependent variable, with age, area, education, and marital status serving as independent variables, as shown in Table 4, Model 1. The standardized beta coefficients for age (0.218), area (−0.99), education (0.214), and marital status (−0.109) indicate a nonsignificant regression, as *p* < 0.05. No significant correlations were found between the dependent and independent variables. However, the positive standardized beta values suggest that age and education are associated with a higher prevalence of HIV.

The second linear regression analysis uses the unsafe-sex score as the dependent variable, with age, area, education, and marital status serving as independent variables, as shown in Table 4, Model 2. The standardized beta coefficients for age (0.005), area (−0.026), education (−0.097), and marital status (−0.057) indicate a nonsignificant regression, as *p* < 0.05. However, a significant negative correlation was found with marital status, suggesting that unmarried individuals have a higher prevalence of HIV due to unsafe sexual practices.

The third linear regression uses the score for sharing syringes and needles as the dependent variable, while age, area, education, and marital status serve as independent variables, as shown in Table 4. The standardized beta scores are as follows: age (0.151), area (−0.0297), education (0.214), and marital status (−0.109). Since *p* < 0.05 indicates nonsignificant regression, it suggests that these variables do not significantly predict the dependent variable. However, a significant correlation was found between sharing syringes and needles and area, indicating that certain regions have a low prevalence of HIV.

The fourth linear regression model uses the drug user score as the dependent variable, while age, area, education, and marital status serve as independent variables, as shown in Table 4 (Model 4). The standardized beta coefficients for age (−0.160), area (−0.298), education (0.200), and marital status (0.107), with *p* < 0.05, indicate a nonsignificant regression. However, a sufficiently strong correlation exists among drug users, suggesting that certain areas with high drug use are associated with elevated HIV prevalence.

## 4. Discussion

Our study indicates a high prevalence of HIV among unmarried individuals, with a significance level of *p* < 0.01. Similarly, the sharing of syringes and needles (0.73) and drug use (0.061) are associated with unsafe sexual practices. A notably strong positive correlation exists between drug use and the sharing of syringes and needles (0.867^∗∗^), and this relationship is highly significant (*p* < 0.01). There is also a slight positive association between drug users (0.138) and blood transfusions, as well as the sharing of syringes and needles (0.73). Although this correlation is minor, it is still noteworthy. In addition, we found that certain populations exhibit a high prevalence of HIV due to a substantial number of drug users. The transmission of HIV through blood transfusions remains a concern, particularly given the inadequate screening practices; thus, the need for universal screening is imperative. The first United Nations (UN) held the first ever General Assembly Special Session (UNGASS) blood safety indicator was developed and distributed in 2006 to assess the advancements that have been achieved in HIV blood screening [9, 10]. The testing of all donated blood for HIV and the establishment of robust quality assurance processes are both crucial; 91 countries have already taken steps in this direction. Conversely, 34 countries have acknowledged that they did not screen all donated blood for HIV by quality requirements, while another 67 countries have failed to provide statistics regarding their screening practices [11]. The intravenous injection of infected blood or blood components is a highly effective method of pathogen transmission [12, 13]. Since the introduction of diagnostic antibody testing, the transmission of human immunodeficiency virus through contaminated blood and blood products has significantly decreased in most affluent countries and even in some developing nations' healthcare systems [14]. Nosocomial infections in Eastern Europe and among individuals who use intravenous drugs illustrate how quickly HIV can spread, even when contaminated injection equipment does not contain a substantial amount of blood [15, 16]. The reason for this is that the same individuals also serve as vectors for the transmission of HIV. Needlesticks and contaminated blood transfusions are the primary types of exposures that can pose a danger; however, there are many other potential exposure risks. When a sharp instrument contaminated with blood or fluids from a jet injector is used, there is a possibility that bacteria may come into contact with the skin [17, 18]. However, the amount of contaminated blood supplied is primarily regulated by the route of exposure. The infectious characteristics of pathogens are the main factors that determine the concentration of these pathogens [19]. The conclusions of the previous research about the frequency of injection drug preparation equipment (IDPE), sharing are confirmed by our findings. It is quite plausible that the risk of HIV transmission is the impetus for this decision, taking into consideration the fact that IDPE is not well recognized among HIV/AIDS disease patients [20, 21]. Those individuals who stated that they would not share needles or syringes with others were more likely to participate in daily injection drug paraphernalia exchange. Due to the well-documented risks associated with the transmission of infectious diseases such as HIV and hepatitis C, the practice of sharing syringes and needles is highly unlikely to occur [22]. Exchanges of needles and syringes, which occur more frequently than those involving IDUs, are associated with a heightened risk of HIV infection [22, 23]. The consistent use of IDPEs, on the other hand, may increase the cumulative risk. This cumulative risk is anticipated to be greater when individuals in a group share intravenous catheters and syringes (IDPE) more frequently than when they share needles and syringes [24, 25]. When evaluating the potential risks associated with sharing needles and syringes, it is reasonable to assume that individuals will only engage in this behavior with partners whom they believe share similar viewpoints. In Kathmandu, Nepal, and Lahore, Pakistan, there is a prevalent practice of sharing injection equipment among individuals who use injectable drugs [26–28]. This is primarily due to the fact that these individuals are facing significant financial difficulties. It is important to note that the cost of a single needle and syringe in India is approximately 10 cents in U.S. currency [29, 30]. It is common for authorities to prohibit individuals who inject drugs from accessing the needles required for drug use. The strength of this study lies in our ability to identify the primary factor that significantly contributes to HIV infection: the sharing of needles and syringes.

### 4.1. Strength of the Study

The data collected from patients with HIV infections offer valuable insights into how risky behaviors contribute to the transmission of the virus. This information was gathered from a hospital where patients were diagnosed using ELISA and PCR methods, ensuring the accuracy of the data. The centralized hospital data allow us to examine potential infection pathways and evaluate hospital interventions. Furthermore, the information on HIV infections among these patients can assist in minimizing transmission through public health interventions and educational programs aimed at HIV eradication.

### 4.2. Limitation of the Study

The limitation of this study is that the study was conducted on small samples; only 67 patients agreed to participate in the study. The females were excluded from the study. The data are observational, so genomic data are also needed to explore the research further. The data were collected from a single medical hospital.

## 5. Conclusion

HIV/AIDS is a rapidly spreading epidemic in Pakistan. Several factors contribute to this issue, including the sharing of needles and syringes, blood transfusions, unprotected sex, sex work, and mother-to-infant transmission, among others. Our study indicates that HIV is easily transmitted among drug users who share syringes and needles. Notably, there is a remarkably strong positive correlation (*r* = 0.867^∗∗^) between drug use and the sharing of syringes and needles, which is highly significant (*p* < 0.01). In addition, our research reveals that certain regions exhibit a high prevalence of HIV due to a large population of drug users. An interesting finding of this study is that we observe a significant correlation between HIV prevalence in unmarried HIV-positive patients.

## Figures and Tables

**Table 1:** The demographic data of HIV-positive patients.

Marital status	Unmarried	24	35.8%
	Married	43	64.2%

Education	No school	8	11.9%
	Primary school	29	43.3%
	High school	22	32.8%
	F.sc	8	11.9%

Area	Azad Jammu Kashmir	11	16.4%
	Gilgit Baltistan	13	19.4%
	KP	9	13.4%
	Sind	21	7.5%
	Punjab	5	31.3%
	Balochistan	8	11.9%

Age	> 20 years	4	5.9%
	> 40 years	46	68.6%
	> 60 years	16	23.8
	< 60 years	1	1.4%

Family income	➢ 100$	41	61.1%
	➢ 150	17	25%
	➢ 200	9	13.43%

**Table 2:** Bivariate analysis of factors associated with blood transfusion, unsafe sex, sharing syringes and needles, and drug users regarding HIV.

Variables	Blood transfusion	Unsafe sex	Sharing syringes and needles	Drugs user
*Marital status (N)*				
Unmarried (24)	1.2500 ± 0.9890	0.7500 ± 0.60792	1.5417 ± 0.72106	1.0000 ± 0.78019
Married (43)	1.3721 ± 0.57830	0.1860 ± 0.50028	1.5714 ± 0.91446	0.9762 ± 1.11504
*P* value	0.525	0.00	0.82	0.927

*Area*				
AJK	1.7273 ± 0.64667	0.4545 ± 0.82020	2.1818 ± 1.25045	1.5455 ± 1.36848
Gilgit Baltistan	1.0000 ± 0.40825	0.3846 ± 0.65044	1.6154 ± 1.0000	1.0000 ± 0.81650
KP	1.2222 ± 0.83333	0.4444 ± 0.52705	1.6667 ± 1.0000	1.4444 ± 1.101,379
Punjab	1.4286 ± 0.00000	0.3810 ± 0.58959	1.3000 ± 0.73270	0.7000 ± 0.97872
Sind	1.0000 ± 0.00000	0.4000 ± 0.54772	1.2000 ± 0.44721	0.4000 ± 0.54772
Balochistan	1.3750 ± 0.74647	0.2500 ± 0.46291	1.3750 ± 0.51755	0.7500 ± 0.46291
*P* value	0.204	0.988	0.088	0.097

*Education level*				
High school	1.1364 ± 0.56023	0.4545 ± 0.67098	1.5714 ± 0.74642	1.0000 ± 1.00000
Primary school	1.3793 ± 0.94165	0.3013 ± 0.54139	1.7241 ± 1.03152	1.0345 ± 1.20957
No school	1.5000 ± 0.53452	0.5000 ± 0.75593	1.3750 ± 0.35355	0.7500 ± 0.46291
FSC	1.5000 ± 0.74647	0.3750 ± 0.51755	1.1250 ± 0.84360	1.0000 ± 0.53452
*P* value	0.496	0.801	0.311	0.919

**Table 3:** Correlation of continuous variables with the blood transfusion, unsafe sex, sharing syringes and needles, and drug users regarding HIV.

Correlations		Blood transfusion	Unsafe sex	Sharing syringes and needles	Drug users
Blood transfusion	Pearson correlation	1	−0.086	0.138	0.130
	*P* value		0.491	0.271	0.299
	*N*	67	67	66	66

Unsafe sex	Pearson correlation	−0.086	1	0.073	0.061
	*P* value	0.491		0.560	0.627
	*N*	67	67	66	66

Sharing syringes and needles	Pearson correlation	0.138	0.073	1	0.867^∗∗^
	*P* value	0.271	0.560		0.000
	*N*	66	66	66	66

Drug user	Pearson correlation	0.130	0.061	0.867^∗∗^	1
	*P* value	0.299	0.627	0.000	
	*N*	66	66	66	66

^∗∗^Correlation is significant at the 0.01 level (2-tailed).

**Table 4:** Multivariable analysis.

Variable	Unstandardized beta	Standardized beta	*t*-value	*P* value
*Model 1: Linear regression taking the blood contamination as the dependent variable*				
Age	0.015	0.218	1.454	0.151
Area	−0.047	−0.099	−0.796	0.429
Education	0.165	0.214	1.711	0.092
Marital status	−0.025	−0.016	−0.109	0.913

*Model 2: Linear regression taking the unsafe-sex as the dependent variable*				
Age	0.000	0.005	0.035	0.972
Area	−0.010	−0.026	−0.228	0.820
Education	−0.040	−0.064	−0.551	0.584
Marital status	−0.573	−0.460	−3.295	0.002

*Model 3: Linear regression taking the sharing syringes and needles score as the dependent variable*				
Age	0.011	0.151	1.021	0.311
Area	−0.158	−0.297	−2.423	0.018
Education	−0.084	−0.097	−0.786	0.435
Marital status	−0.099	−0.057	−0.383	0.703

*Model 4: Linear regression taking the HIV score as the dependent variable*				
Age	−0.014	−0.160	−1.068	0.290
Area	−0.188	−0.298	−2.399	0.019
Education	0.021	0.020	0.159	0.874
Marital status	0.220	0.107	0.711	0.480

*Note:* Standard beta represents the change in dependent variables in a mean of standard deviations unit concerning change in the standard deviation of independent variables. It allows the comparison of the different predictors regarding their scales.

## Data Availability

The data used to support the findings of this study are included within the article.
